# Antiphospholipid Syndrome—Diagnostic and Methodologic Approach

**DOI:** 10.3390/metabo15080500

**Published:** 2025-07-27

**Authors:** Agata Stańczewska, Karolina Szewczyk-Golec, Iga Hołyńska-Iwan

**Affiliations:** 1 L. Rydygier Provincial Integrated Hospital in Torun, ul. Świętego Józefa 53-59, 87-100 Torun, Poland; agatastanczewska@wp.pl; 2 Department of Medical Biochemistry and Biology, Faculty of Medicine, Ludwik Rydygier Collegium Medicum in Bydgoszcz, Nicolaus Copernicus University in Torun, 87-100 Torun, Poland; 3 Department of Pathobiochemistry and Clinical Chemistry, Faculty of Pharmacy, Ludwik Rydygier Collegium Medicum in Bydgoszcz, Nicolaus Copernicus University in Torun, 87-100 Torun, Poland

**Keywords:** antiphospholipid antibodies, antiphospholipid syndrome, catastrophic APS, lupus anticoagulant

## Abstract

Antiphospholipid syndrome (APS) is an autoimmune disorder characterized by venous and arterial thrombosis and obstetric complications, driven by antiphospholipid antibodies (APLAs). This review synthesizes the latest advancements and current understanding, diagnosis, and treatment of APS. APLAs, including lupus anticoagulant (LAC), anticardiolipin (aCL), and anti-β2-glycoprotein I (aβ2-GPI), interfere with coagulation and endothelial function, as well as with placental health. APS can be primary or secondary; it is often associated with systemic autoimmune diseases like lupus. The pathogenesis of APS remains only partially understood. APLAs promote thrombosis through endothelial damage, platelet activation, and inflammatory signaling pathways. Laboratory diagnosis relies on persistent positivity for APLAs and LAC through tests like ELISA and clotting assays, following a three-step confirmation process. New integrated test systems have been introduced to improve standardization. Classification criteria have evolved, with the 2023 EULAR-ACR criteria providing a weighted, domain-based scoring system, enhancing diagnostic precision. Catastrophic APS (CAPS) is a severe, rare manifestation of APS, characterized by multi-organ failure due to rapid, widespread microthrombosis and systemic inflammation, which requires urgent anticoagulation. Seronegative APS is proposed for patients with clinical features of APS but negative standard antibody tests, possibly due to non-criteria antibodies or transient immunosuppression. Treatment primarily involves long-term anticoagulation with vitamin K antagonists; direct oral anticoagulants are generally not recommended. APS diagnosis and management remain complex due to clinical heterogeneity and laboratory challenges. Continued refinement of diagnostic tools and criteria is essential for improving outcomes in this life-threatening condition.

## 1. Introduction

Antiphospholipid syndrome (APS) is a complex autoimmune disorder with a pathomechanism involving immunological, vascular, and platelet modifications caused by circulating antiphospholipid antibodies (aPL) [1,2,3,4,5,6,7,8]. Those antibodies are a predisposing factor of the disease and can initiate thrombosis. APS is a systemic disease clinically characterized by thrombosis (both venous and arterial) leading to thromboembolic changes and, in women, additionally to obstetric complications [4,7,9,10]. The disease affects the vascular beds of arteries of all sizes, as well as veins and microvessels [1,2,3,4,5,6,11]. However, venous thrombosis is a dominant form [1,12]. It most often affects the veins of the lower limbs, frequently coexisting with pulmonary embolism. Arterial thrombosis affects the coronary or cerebral arteries, leading to myocardial infarction or stroke. However, changes could also lead to nephropathy [13] and retinopathy [8]. Sometimes the thrombus affects veins in so-called atypical locations, which include mainly cerebral veins and venous sinuses of the meninges [14], splanchnic veins, portal vein, hepatic veins (Budd–Chiari syndrome), and mesenteric veins [15].

Diagnosis of APS is a clinical problem, despite existing recommendations and a wide selection of laboratory tests. This review aims to synthesize current knowledge on the pathomechanisms underlying the development of APS, including catastrophic APS (CAPS), present the latest clinical recommendations, and outline the principles and applications of laboratory methods used in evaluating patients with APS.

## 2. Antiphospholipid Antibodies

APL is a heterogeneous group of autoantibodies directed against various antigens. They include autoantibodies against plasma proteins such as β2-glycoprotein I (β2GPI) and domain I of β2GPI, prothrombin, annexin V, protein C, protein S, factor XII, thrombin, kininogens, and others [3,7,15]. APL also demonstrates affinity for negatively charged phospholipids of the cell membrane (cardiolipin, phosphatidylcholine, phosphatidylethanolamine, phosphatidylserine) and can disrupt the phospholipid’s normal function [3,16,17,18]. These autoantibodies can bind to phospholipid molecules on the surface of the endothelial cells of blood vessels and to phospholipid-binding proteins, including β2GPI with a strong affinity for cardiolipin, prothrombin, annexin 5, and protein C [3,15,16,18,19]. As a result, anticoagulant processes are disturbed. The autoantibodies directly damage the endothelium, leading to the formation of clots in arteries and veins. It results in ischemia of organs, including the placenta [3,16,18]. APL belongs to different antibody classes (IgG, IgM, IgA) and shows different specificities. The most common group includes IgG (IgG2 and IgG4), and the least common, IgA. IgG antibodies show a stronger correlation with thrombosis and miscarriages than IgM antibodies [20]. Antibodies of clinical significance for APS include lupus anticoagulant (LA), anticardiolipin antibodies (aCL), and antibodies against β2GPI (aβ2-GPI). The presence of LA is the strongest risk factor for arterial and venous thrombosis [21]. Some of the aPL produced, as isotypes of IgA anti-domain I β2-GPI, or IgG and IgM anti-phosphatidylserine/prothrombin complex, occur rarely, and thus they are not included in the guidelines for the diagnosis and evaluation of patients with APS [22,23]. However, those autoantibodies might be clinically significant, especially in seronegative cases.

ACL can arise as alloantibodies or autoantibodies [15,16]. In infectious diseases, especially in syphilis, alloantibodies appear that bind to negatively and neutrally charged phospholipids without requiring a protein cofactor. Post-infectious antibodies usually do not exhibit prothrombotic properties and therefore usually do not pose a risk of thromboembolic complications. On the other hand, autoantibodies found in autoimmune diseases bind only to negatively charged phospholipids and require a protein cofactor [15,16].

**Figure 1:**
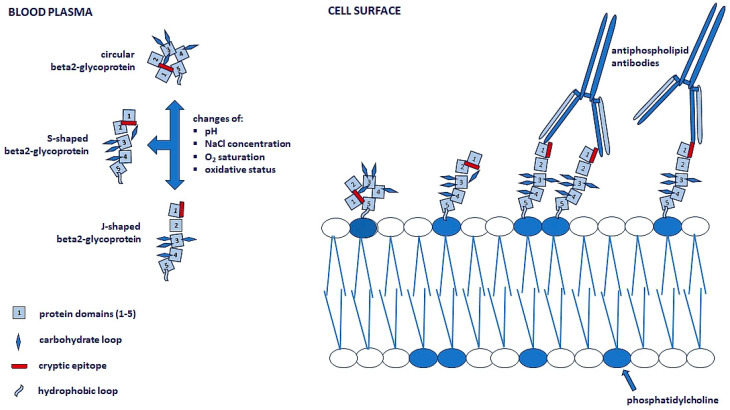
The interaction between β2-microglobulin, phospholipids of plasma membranes, and antiphospholipid antibodies leads to the activation of target cells, including platelets, monocytes, macrophages, neutrophils, and endothelial cells. β2-microglobulin undergoes conformational changes in the blood plasma under the influence of extrinsic and intrinsic factors. In circular and S-shaped forms, it attaches to membrane phospholipids but has no ability to bind to antibodies. Only the J-shaped form has this ability, because this form has a cryptic epitope available for interaction with antibodies [25,26].

The detailed mechanism of autoantibody production in primary APS has not been fully elucidated [3,15,16,24,25,26,27]. Abnormal maturation and differentiation of B cells is the primary cause of the production of these autoantibodies [28]. APL binds to the open and immunogenic molecules of β2-GPI on the endothelial surface (Figure 1), leading to the activation of target cells such as endothelial cells, platelets, monocytes, tumor necrosis factor alpha (TNF-α)-secreting macrophages, and neutrophils (releasing neutrophil extracellular traps (NETs) during NETosis, a specific cell death of neutrophils) [4,29]. Furthermore, this binding increases the expression of the mitogen-activated protein kinase (MAPK) and nuclear factor kappa B (NF-κB) pathways, ultimately leading to thrombosis [4,29].

The results of cohort studies suggest the occurrence of the autoantibodies in 1 to 12% of healthy individuals [30]. The overall incidence of APS is estimated at 50 per 100,000 individuals, with a female-to-male ratio of approximately 5:3.1 [10,31]. The presence of aPL in individuals without clinical symptoms may be the result of neoplastic diseases, infections, vaccinations, or medications taken. Drugs that can stimulate aPL production include procainamide, phenothiazine, hydralazine, chlorothiazide, and oral contraceptives [32]. Drug-induced aPL are usually low-titer and transient. Moreover, aPL are found in 25% of patients with venous thrombosis, 25% of young patients with stroke, 18% of patients with premature coronary atherosclerosis, and approximately 30% of patients with recurrent pregnancy loss [33,34]. In systemic lupus erythematosus (SLE) patients, aPL are diagnosed in 20–60% of cases [34,35].

## 3. Pathophysiology of Antiphospholipid Syndrome

The pathogenesis of thrombosis in APS has not been fully established (Table 1). It is known, however, that aPL plays a role by affecting platelet cell membranes, endothelial cells, and proteins involved in the blood coagulation cascade. The significance of environmental or genetic factors that could stimulate aPL production has also not been established.

The pathomechanism of APS leading to pregnancy loss has also not been explained [36,37]. One of the hypotheses assumes an adverse effect of APS on trophoblast function in early pregnancy, resulting in placental vessel thrombosis [36]. Increasing evidence suggests that the autoantibodies disrupt normal trophoblast biology, leading to inhibition of trophoblast invasion and initiation of an inflammatory response, which in turn may lead to miscarriage [36]. In some cases, familial occurrence of APS has been observed, particularly in association with specific human leukocyte antigen (HLA) histocompatibility markers, including HLA-DR7, DR4, DQw7, and DRw53 [38].

## 4. Varieties of the Antiphospholipid Syndrome

Depending on the presence or absence of autoimmune disease, two forms of APS have been distinguished, namely primary APS (PAPS) and secondary APS (SAPS). PAPS is not accompanied by autoimmune disease, whereas SAPS is accompanied by systemic connective tissue diseases, infections, neoplasms, immunohematolytic diseases, and others (Table 2).

## 5. Classification of Antiphospholipid Syndrome

In order to standardize the classification, criteria for the diagnosis of APS were introduced [39,40,41]. The first criteria were developed during the Sapporo conference and published in 2006 [5]. It was established that to qualify for APS, it was necessary to meet one clinical criterion and one laboratory criterion. The clinical criterion included venous, arterial, or small blood vessel thrombosis, and additionally, in women, obstetric complications. The laboratory criterion included the presence or absence of LA and/or moderate or high titer aCL and/or aβ2-GPI antibodies in the IgG or IgM class [25,27]. However, these guidelines did not address many important clinical and laboratory symptoms, such as thrombocytopenia, hemolytic anemia, valvular heart disease, and nephropathy, that coexist with APS and may also occur without a positive LA result [8,23]. Moreover, since the criteria were published, new types of aPL, strongly correlated with thrombotic symptoms of the syndrome, have been detected. Among them, it is worth mentioning especially the antibodies against domain I of β2-GPI (aDI), which are considered the most pathogenic type of aPL [27]. Antibodies directed against phosphatidylserine/prothrombin complexes (aPS/PT) are strongly associated with the detection of LA [17]. An independent role of aPL in the IgA class was also sought. It is known that the risk of thrombotic episodes might have various manifestations, depending on the detected antibodies, their titer, and the transient or permanent nature. Thrombotic symptoms of origins other than autoantibody-related should also be considered.

Based on new scientific data, an attempt was made to develop new classification criteria using the methodology implemented by the European Alliance of Associations for Rheumatology (EULAR) [41]. In 2023, new classification criteria for the APS were published, in accordance with the unified EULAR methodology, developed by a team of experts, including members of the American College of Rheumatology (ACR) and EULAR (Table 3) [41].

The basis for further classification of patients is the fulfillment of one initial criterion: one documented clinical criterion, then additional clinical symptoms and laboratory criteria, divided into 8 domains, are considered. Additionally, the symptoms and test results included in these domains were assigned a certain weight, defined by the number of points [6,10]. In order to diagnose APS, meeting the initial criterion and scoring at least 3 points from the clinical domains and 3 points from each of the laboratory domains is required. Current recommendations are characterized by 99% specificity and 84% sensitivity for detecting APS [39,40,41]. Additionally, they also serve to select homogeneous groups of patients in clinical trials.

## 6. Catastrophic Antiphospholipid Syndrome

CAPS, or Asherson syndrome, is a rare clinical form of APS. Its characteristic feature includes a sudden onset and rapidly progressing multi-organ failure. It develops in less than 1% of patients with APS but is characterized by high mortality, reaching 37%, caused by multi-organ failure [9,39]. CAPS most often develops in people diagnosed with APS who have experienced an additional factor initiating an intense generalized reaction, such as infection or surgery. As a result of the formation of clots in the microcirculation of the vascular bed, disseminated thrombosis and symptoms of systemic inflammatory response (SIRS) develop, leading to multi-organ failure [36]. Thrombosis mainly affects the blood vessels of the abdominal cavity, kidneys, lungs, and nervous system. In patients with CAPS, simultaneous involvement of several organs in a short time and signs of occlusion of small blood vessels are observed. Laboratory test results confirm the presence of aPL, usually in high titers [9]. The basis of the CAPS is the rapid appearance of disorders of hemostasis, both coagulation and fibrinolysis, caused by aPL, but most of the inducing factors remain unknown.

The clinical manifestations of CAPS depend on factors such as which major organ is affected first, the degree of thrombosis, and the development of SIRS. The first clinical symptoms in the course of CAPS include pulmonary complications (24% of patients), neurological symptoms (18% of patients), and renal symptoms (18% of patients) [9]. Thrombotic complications may also affect the adrenal glands, spleen, intestines, and their vessels, causing abdominal pain of unclear etiology. Renal involvement in the course of the disease is found in 71% of patients, manifested by acute renal failure, severe hypertension, proteinuria, and hematuria [36]. Acute respiratory distress syndrome in the form of pulmonary embolism, bleeding, and pulmonary edema occurs in about 64% of patients, where dyspnea is the dominant clinical symptom. The central nervous system (CNS) is affected in 62% of patients, most often manifested by cerebral venous obstruction, seizures, infarctions, and encephalopathy [36]. Myocardial symptoms affect 51% of patients in the form of valvular defects, while myocardial infarction affects 25% of patients. In about 50% of cases, the occlusion of small blood vessels causes skin complications manifested as livedo reticularis, purpura, and/or skin necrosis [37]. Most CAPS patients have microangiopathy, i.e., occlusive vascular disease affecting mostly small vessels of various organs, mainly kidneys, lungs, brain, heart, and liver, and only a small proportion have large-vessel thrombosis, typical of the common APS [37]. Early diagnosis of CAPS reduces the risk of mortality in patients by about 30%. Therefore, the correct treatment of this syndrome consists of early diagnosis and aggressive anticoagulant therapy. Due to the heterogeneity of the clinical picture, in September 2002, during the workshop preceding the 10th International Congress on aPL, consensus criteria for the diagnosis and classification of CAPS were established (Table 4) [9,39].

Due to the rare but life-threatening occurrence of CAPS, high clinical vigilance is required. There are clinical situations in which symptoms characteristic of APS are noted, but the presence of aPL, included in the criteria, cannot be demonstrated. Therefore, it has been proposed to create a category of the so-called seronegative APS (SN-APS). It is necessary to take into account the high heterogeneity of antibodies. Thus, it should be considered that the diagnostic criteria for APS do not include all types of aPL that might be present in the blood plasma of patients. In some patients who test negative for aCL and aβ2-GPI IgG and IgM antibodies, these antibodies may be produced in the IgA class [26]. Moreover, antibodies against phosphatidylserine, phosphatidic acid, or phosphatidylinositol may be present. The phenomenon of so-called transient seronegativity caused by the use of corticosteroids or other immunosuppressive drugs should also be considered [10,27].

## 7. Laboratory Diagnostics of Antiphospholipid Syndrome

Laboratory diagnostics are a key element in the diagnosis of APS, enabling, among other things, detecting the presence of aPL and their titer, confirming the persistent presence of aPS, and assessing the risk of thrombosis and other complications. To get reliable results, appropriate preparation of the patient for the test, up-to-date information about the patient’s health status, proper sample handling, and choosing the right aPL detection techniques are of first importance. The proper determination of aPL translates into the correct APS diagnosis and assessment of the risk of complications [39,40,41].

### 7.1. Standard Laboratory Criteria

According to the current recommendations of the Scientific and Standardization Committee of the International Society on Thrombosis and Hemostasis (ISTH) from 2020 [42] and 2025 [43], the diagnosis of APS consists of determining the titer of aCL in the IgG and IgM classes, aβ2-GPI in the IgG and IgM classes, and detecting LA. Positive LA means a presence of heterogeneous antibodies that prolong laboratory results of phospholipid-dependent coagulation tests, i.e., tests based on activated partial thromboplastin time (APTT) and dilute Russell viper venom test (dRVVT) [16,30,44,45]. Therefore, the detection of LA is performed using coagulation tests, based on the phospholipid-dependent plasma clotting times in platelet-poor citrated plasma, double-centrifuged to avoid the influence of phospholipids released from platelets. According to recommendations, the test involves the use of two screening tests due to differences in their sensitivity and specificity, namely APTT with a reagent with low phospholipid content and dRVVT [44,45]. Diatomaceous earth (silica) is the preferred clotting activator in the APTT test, although the use of ellagic acid is also accepted [3,30]. To confirm the presence of an anticoagulant that is specifically associated with prothrombotic tendency, a three-step procedure is used [3,16,46]: (1) determination of APTT time using two tests from the patient sample; prolonged clotting time allows for the next step; (2) performing a correction test (so-called mixing test): mixing the same proportion of the patient’s plasma and normal plasma to check whether the prolonged APTT time will shorten or remain prolonged; if the tested time is shortened, it will indicate a deficiency of coagulation factors, whereas if it remains prolonged, it will confirm the presence of an inhibitor, which may be, among other things, LA; (3) confirmation test, consisting of proving the shortening of the prolonged clotting time in the patient sample after the addition of excess phospholipids derived, for example, from liposomes containing phosphatidylserine, from rabbit brain, alternately frozen and thawed platelets, or hexagonal phospholipids; the shortening of the time indicates the phospholipid-dependent specificity of the phenomenon and indicates the presence of LA [46]. In order to confirm the persistent or chronic presence of LA, it is absolutely necessary to repeat the test in the patient’s test after at least 12 weeks [30,47,48].

### 7.2. Lupus Anticoagulant Testing

The current recommendations mention the discussion on the so-called integrated tests, which simplify the three-step procedure for determining the presence of LA [3,30,49]. The integrated tests allowed the introduction of the LA examination to laboratories with less experience in APS diagnostics. These tests available on the market include a screening test and a confirmation test. These tests require testing of plasma samples with dual APTT and dRVVT tests. The first test should be used at low (screening) phospholipid concentrations, and the second test at high (confirmatory) phospholipid concentrations [3]. Based on the coagulation times, coefficients are calculated (for the screening and confirmation tests), and finally, the total/normalized ratio (Total Ratio) is calculated. Based on the obtained value, the test can be interpreted as positive or negative. The higher the coefficient, the greater the probability of a positive LA result [49]. The coefficient values are provided by the test manufacturer. Commercially available integrated tests differ in the clotting activator. Diatomaceous earth is used in Silica Clotting Time (SCT). Two tests are available, SCT Screen and SCT Confirm, which are reagent kits designed to simplify and standardize the detection of LA in clinical evaluations. SCT Screen is poor in phospholipids, making it sensitive to LA. The additional amount of phospholipids in SCT Confirm neutralizes LA to shorten the clotting time. Diatomaceous earth in the presence of calcium directly activates the intrinsic coagulation pathway. Therefore, SCT Screen and SCT Confirm are resistant to factor VII deficiencies or inhibitors. The use of screening and confirmation ratios allows SCT to be insensitive to warfarin-treated samples. As a result, SCT Screen and SCT Confirm are more specific tests for LA than APTT [16,30]. A normalized ratio value >1.16 indicates the presence of LA. HemosIL dRVVT Screen and dRVVT Confirm are tests using diluted Russell’s viper venom. The dRVVT Screen is low in phospholipids, making it sensitive to LA, while the dRVVT Confirm neutralizes LA and provides a shorter clotting time if LA is present. A normalized ratio value >1.2 indicates the presence of LA. The combination of SCT/dRVVT tests has been shown to increase the detection of LA [3,30]. The diagnosis of LA is very extensive and difficult due to the great heterogeneity of aPL, which translates into difficulties in establishing uniform laboratory standards for LA detection, determining its potency, and methods of determination [3].

### 7.3. ACL and aβ2-GPI Detection

ACL and aβ2-GPI are detected using immunochemical methods such as ELISA (enzyme-linked immunosorbent assay), chemiluminescence (CLIA), or automated addressable laser bead immunoassay (ALBIA) [18]. In the case of solid-phase antibody detection, the lack of influence of a patient’s anticoagulation is an advantage. A cut-off value of aPL concentration of diagnostic significance has been established as follows: the antibody concentration is diagnostically significant if it is higher than 40 GPL or MPL units or is above the 99th percentile [1,50]. To be more specific, the aPL result (in both classes) indicates APS if it is positive (range 40–79.9) or highly positive (above 80) in GPL or MPL units, at least twice with an interval of 12 weeks, when determined using the ELISA or CLIA, or ALBIA method [16,18,30]. However, commercially available ELISA or CLIA tests do not have standardized cut-off values, but only those proposed by the manufacturer [30,50].

**Figure 2:**
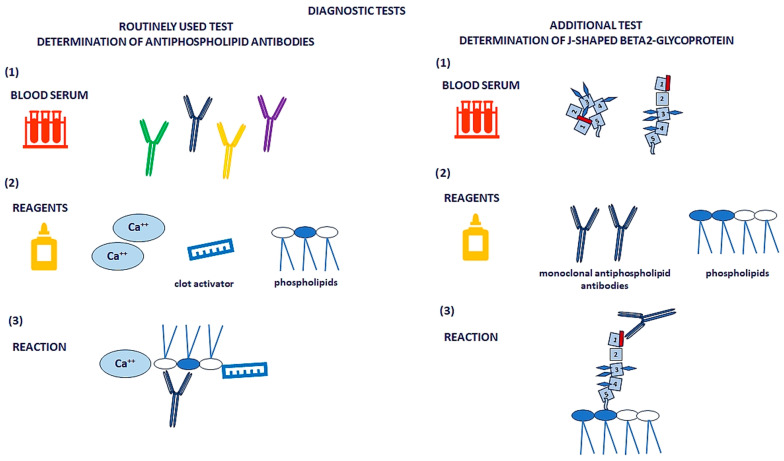
Comparison of methods for detecting antiphospholipid antibodies on the example of β2-glycoprotein. Conventional tests for antiphospholipid antibodies use a mixture of phospholipids, calcium ions, and coagulation activators to detect a broad range of antibodies in patient serum. These tests identify both clinically significant and insignificant antibodies and often produce cross-reactions. As such, they are primarily used for screening purposes. In contrast, modern assays incorporate phospholipids and highly specific monoclonal antibodies that bind to a single antigenic site on β2-glycoprotein, but only when its conformation reflects in vivo conditions that lead to antibody production. These advanced tests detect only clinically relevant antibodies and are used for the definitive diagnosis of antiphospholipid syndrome.

Figure 2 presents the details of routinely used and additional, more advanced tests that enable the determination of β2GPI of J-shape, which is involved in the development of APS.

### 7.4. Risk Stratification and Interpretation

The laboratory assessment of aPS of different types enables the proper diagnosis and approximation of the complication risk. Patients with triple positivity are characterized by a higher risk of venous and arterial thrombosis than patients with double positivity (Table 5). Triple positivity indicates the positive results of all of the above-mentioned laboratory parameters, namely LA, aCL, and aβ2-GPI or aPS/Pt. Additionally, the Global APS Score (GAPSS) has been introduced. GAPSS is an effective scale for estimating thromboembolic events and/or obstetric failures because, in addition to the presence of aPL, it also takes into account the presence of hyperlipidemia and hypertension [49].

It should be emphasized that, in order to properly assess the risk of thrombotic complications in the course of APS, the above-mentioned antibodies should be tested, but the clinical probability of APS should be taken into account. Uncritical testing of LA and other autoantibodies, including the frequently prescribed antinuclear antibodies, may lead to accidental detection of their presence, which stigmatizes the examined patient and is usually not associated with any specific clinical significance [3,16]. Indications for the determination of LA are presented in Table 6.

### 7.5. Pre-Analytical Considerations

Appropriate preparation of the patient for aPL determination is extremely important because clinical situations related to inflammation or therapy used may cause false-positive or false-negative results. During acute inflammation or an acute thrombotic episode, in which the concentration of acute-phase proteins increases, it is not recommended to perform the tests [3,10,16]. Anticoagulant therapy is another cause of false-positive or false-negative results. Patients taking anticoagulants must be adequately prepared to reliably determine the presence or absence of LA while ensuring the continuity of anticoagulation therapy. In patients treated with vitamin K antagonists (VKA), interpretation of results is difficult because the basic coagulation tests are initially prolonged [3]. It is recommended to discontinue VKA for 1–2 weeks, replacing VKA with low-molecular-weight heparin (LMWH). In such cases, samples should be taken at least 12 h after the last dose of LMWH, and, simultaneously, antibodies for factor Xa should be measured. LA can also be measured during treatment with direct oral coagulation inhibitors (DOACs), if, based on pragmatism and empirical experience, the patient stops taking DOAC for 48 h or slightly longer in case of impaired renal function [3]. In addition to acute inflammation or anticoagulant treatment, attention should also be paid to pregnant patients. Due to the hemostatic changes that occur during pregnancy, LA should be measured after delivery.

## 8. Treatment of Antiphospholipid Syndrome

### 8.1. General Principles of Treatment

Anticoagulation is the primary treatment for APS. However, it is ineffective in approximately 20–30% of obstetric cases, where pregnancy loss and perinatal complications occur despite treatment, and in over 30% of thrombotic APS cases due to the recurrent thrombotic events [7,38]. Patients with APS and thrombosis are at high risk of recurrent thrombosis; therefore, anticoagulation is recommended for an indefinite period [51,52]. The treatment can be divided into 3 main categories, namely prophylactic, specific, and non-specific treatment. APS patients with unprovoked venous thrombosis should be treated long-term with acetylsalicylic acid (ASA), VKA, or DOACs with a target international normalized ratio (INR) of 2.0–3.0 [40]. The 2019 EULAR recommendations [40] define the treatment method based on the assessment of thrombotic risk in patients with aPL (Table 5). Each of the recommended drug groups has side effects; however, in the case of using DOACs in triple-positive patients, the risk of arterial events increases, which is particularly unfavorable [33,40,53]. DOACs such as rivaroxaban or apixaban should be particularly avoided due to the increased risk of arterial thrombosis and cerebral ischemic episodes [54]. This increased risk was evaluated in the Apixaban for Secondary Prevention of Thromboembolism Among Patients with Antiphospholipid Syndrome (ASTRO-APS) trial [55].

### 8.2. Primary Prevention

Detailed recommendations for primary APS in adult patients are presented in Table 7. Generally, ASA is recommended in low doses after assessing the risk of thrombotic events.

### 8.3. Secondary Prevention

Recommendations for secondary prevention of APS in adults are presented in Table 8. Treatment is based on personalized adjustment of drug doses in relation to INR measurement results, which should have a value of 2.0–3.0.

### 8.4. Obstetric and Catastrophic APS

Recommendations for the prevention of obstetric and catastrophic APS in women are presented in Table 9. Oral coagulation inhibitors are recommended for women with CAPS. However, prophylactic therapy for pregnant, postpartum, and postmenopausal women and before planned gynecological procedures depends on the presence of autoantibodies and previous episodes of thrombosis/thromboembolism [39,40].

Future research directions should include the search for therapies that modify the affinity of autoantibodies to platelets and/or vessel walls, inhibit the cascade of events occurring after the combination of aPL with the antigen, or modify the function of immune cells in terms of autoantibody production.

## 9. Conclusions

Diagnosing the APS is a challenge for clinicians and cooperating laboratory diagnosticians. Correct diagnosis of this syndrome is crucial for the implementation of proper treatment, namely systemic anticoagulation, often continued for the rest of life. The clinical practice of APS diagnosis and treatment is diverse because the disease is rare and the knowledge about the diagnosis, classification, and clinical spectrum is constantly developing. It is worth noting that APS is a disease with increased thrombotic risk. Importantly, in the case of coexistence of other autoimmune diseases, such as SLE, the risk of hemostasis disorders may be significantly increased. Autoimmune diseases constitute an independent risk of thrombosis [40]. During diagnostics, one should be aware of the importance of proper preparation of the patient for tests, conditions of sample collection, and conducting tests referring to clinical symptoms. Particular attention should be paid to the interpretation of the results of basic laboratory tests and aPL tests, along with a detailed assessment of the patient, because such an approach accelerates diagnostics and proper therapy.

## Figures and Tables

**Table 1:** Probable mechanisms responsible for thrombosis in antiphospholipid syndrome [4,35].

**Endothelial** **cell** **dysfunction**	Antiphospholipid antibody-dependent eNOS inhibition
	Increased expression of adhesion molecules: ICAM-1, VCAM-1, E-selectin, and others
	Increased adhesion of leukocytes to the endothelium
**Platelet** **activation**	Increased thromboxane A2 production
	Increased platelet activation leading to increased glycoprotein IIb/IIIa expression
	Disturbance of vWF-dependent platelet adhesion
	Increased platelet-derived microparticle formation
**Activation of** **the complement** **system**	Activation of C3, C5 components, and deposition of complement components
**Cellular** **Inflammatory** **processes**	Increased expression of TF on monocytes and monocyte-derived microparticles
	Increased release of IL-8
	Release of NETs
**Impairment of** **anticoagulant** **mechanisms**	Antiphospholipid antibody-dependent anticoagulant properties of annexin V
	Inhibition of the protein C system
	Disturbances of antithrombin function
	TFPI inhibition
**Inhibition of** **fibrinolysis/** **Abnormal clot** **structure**	Inhibition of plasminogen binding, activation, and activity of plasmin
	Increased PAI-1 levels
	Prothrombotic clot phenotype: denser fibrin networks, low permeability, reduced susceptibility to lysis

Abbreviations: eNOS—endothelial NO synthase, ICAM-1—intercellular adhesion molecule 1, IL-8—interleukin 8, NET–neutrophil extracellular trap, PAI-1—plasminogen activator inhibitor-1, TF—tissue factor, TFPI—tissue factor pathway inhibitor, VCAM-1—vascular cell adhesion molecule 1, vWF—von Willebrand factor.

**Table 2:** Diseases that may be associated with the presence of antiphospholipid antibodies and antiphospholipid syndrome.

**Autoimmunological** **disorders**	Systemic lupus erythematosus Rheumatoid arthritis Systemic sclerosis S-syndrome Takayasu’s disease Dermatomyositis Autoimmune thyroiditis
**Virological** **disorders**	Human Immunodeficiency Virus Epstein–Barr Virus Hepatitis C Virus Hepatitis B Virus Rubella virus Mumps virus Parvovirus B19
**Bacteriological** **disorders**	Tuberculosis Syphilis Leprosy Rheumatic fever Bacterial endocarditis Klebsiella
**Parasitic** **disorders**	Malaria Toxoplasmosis
**Cancers**	Lungs Colonial Prostate Cervix Liver Kidneys Esophagus Breast
**Hematological** **malignancies**	Myeloproliferative: myeloid leukemias, polycythemia vera, myelofibrosis Lymphoproliferative: B-cell leukemia/lymphoma, non-Hodgkin’s and Hodgkin’s lymphomas Paraproteinemias: multiple myeloma, Waldenström’s macroglobulinemia, monoclonal gammopathies
**Hematological** **disorders**	Sickle cell disease Pernicious anemia Autoimmune thrombocytopenic purpura
**Drug-induced**	Hydralazine Procainamide Quinine Phenothiazine Chlorpromazine
**Others**	Diabetes Inflammatory bowel disease

**Table 3:** Antiphospholipid syndrome classification criteria according to the American College of Rheumatology/European Alliance of Associations for Rheumatology [41].

**ENTERING CRITERIA** **At least one documented clinical criterion**, included in domains D1–D6, **and a positive antiphospholipid antibody (aPL) test result:** lupus anticoagulant, moderate or high titer of anticardiolipin or anti-β2-glycoprotein I antibodies, IgG or IgM class, obtained within 3 years of documented clinical criteria.	
**↓**	
If absent, do not classify as APS, but apply additional criteria.	
**↓**	
**ADDITIONAL CLINICAL AND LABORATORY CRITERIA** Do not count a clinical criterion if there is a more probable cause than APS. Only the criterion with the highest weighting (score) should be counted within each domain	
**Clinical criteria and domain**	**Weight**
**D1. Large-vessel VTE**	
- in a patient with a high-risk profile	**1**
- in a patient without a high-risk profile	**3**
**D2. Large-vessel arterial thrombosis**	
- in a patient with a high cardiovascular risk profile	**2**
- in a patient without a high-risk profile	**4**
**D3. Microvessels**	
Suspected one of the following:	**5**
- livedo racemosa in physical examination	
- livedoid vasculopathy in physical examination	
- acute/chronic aPL-related nephropathy in physical or laboratory examination	
- intravesicular hemorrhage in physical or imaging examination	
Established diagnosis of one of the following:	**5**
- livedoid vasculopathy in physical examination or histopathology	
- acute/chronic aPL-related nephropathy	
- intravesicular hemorrhage in BAL or histopathology	
- myocardial disease imaging or histopathology	
- adrenal hemorrhage imaging or histopathology	
**D4. Obstetric complications**	
- consecutive **lost ≥ 3 pregnancies** at the (pre)embryonic stage (<10 weeks) or	**1**
fetal death (10 weeks 0 days—15 weeks 6 days)	
- **fetal death** (16 weeks 0 days—33 weeks 6 days) without the presence of severe preeclampsia or severe placental insufficiency	**1**

- **severe preeclampsia** (<34 weeks 0 days) **or severe placental insufficiency**	**3**
(<34 weeks 0 days) with or without fetal death	
- **severe preeclampsia** (<34 weeks 0 days) **and severe placental insufficiency**	**4**
(<34 weeks 0 days) with or without fetal death	
**D5. Heart valves**	
- thickening	**2**
- vegetations	**4**
**D6. Hematology**	
- thrombocytopenia (20–130 × 10^9^/L)	**2**
**Laboratory criteria and domain**	**Weight**
**D7. aPL testing by functional coagulation test (LA test)**	
- positive LA once occasionally	**1**
- positive LA permanently	**5**
**D8. aPL testing by solid-phase ELISA (aCL or a-β2GPI) is permanently present**	
- moderately (40–79U)/highly (≥80U) positive **aCL and/or a-β2GPI M** class	**1**
- moderately positive **aCL and/or aβ2-GPI IgG** class	**4**
- highly positive **aCL or aβ2-GPI IgG** class	**5**
- highly positive **aCL and aβ2-GPI IgG** class	**7**
**↓**	
**SCORE** For research purposes, classify as APS if at least **3 points from clinical domains and 3 points from laboratory domains** are collected	

Abbreviations: APS—antiphospholipid syndrome, aCL—anticardiolipin antibodies, a-β_2_GPI—anti-β2-glycoprotein I antibodies, BAL—bronchoalveolar lavage, LA—lupus anticoagulant, ELISA–enzyme-linked immunosorbent assay, VTE—venous thromboembolism.

**Table 4:** Preliminary criteria for the classification of catastrophic antiphospholipid syndrome (CAPS) [39].

**Criteria** 1. Evidence of involvement of ≥3 organs, systems, or tissues. Clinical evidence of vascular occlusion is usually required, confirmed by imaging studies if possible. Renal involvement is defined as a 50% increase in creatinine, severe systemic hypertension (>180/100 mmHg), or proteinuria (>500 mg/24 h). 2. Development of symptoms concurrently or in less than a week. 3. Histopathological confirmation of small-vessel occlusion in at least one organ or tissue. Histopathological confirmation must include significant evidence of thrombosis, although vasculitis may occasionally be present. 4. Laboratory confirmation of aPL (lupus anticoagulant or anticardiolipin antibodies). If the patient has not previously been diagnosed with APS, laboratory confirmation requires at least two detections of aPL within 6 weeks (not necessarily at the time of a relapse), according to the proposed initial criteria for classification of definite APS.
**A definite diagnosis of CAPS** All 4 criteria.
**Probable CAPS** ▪ All 4 criteria, but only with involvement of 2 organs, systems, or tissues. ▪ All 4 criteria, except laboratory confirmation within 6 weeks or more, due to the early death of the patient, who had not been previously tested for aPL before the CAPS episode. ▪ Criteria 1, 2, and 4. ▪ Criteria 1, 3, and 4, and the development of a third symptom within >1 week but <1 month, despite anticoagulation.

Abbreviations: APS—antiphospholipid syndrome, aPL—antiphospholipid antibody.

**Table 5:** Assessment of thrombotic risk in patients with antiphospholipid (aPL) antibodies [10,49].

Thrombotic Risk Level	Type of aPL	Presence of Antibodies	Titer of Antibodies
High	Tripositivity or two types of antibodies: anticardiolipin (aCL) IgG or IgM, anti-β2-glycoprotein (aβ2-GPI) IgG or IgM, antiphosphatydylserine/prothrombin complex (aPS/Pt) IgG or IgM, including lupus antibodies (LA)	Long-term	High
Moderate	Two types of antibodies: aCL IgG or IgM, anti-β2-glycoprotein IgG or IgM, ACL, and aβ2-GPI aPS/Pt IgG or IgM but without LA	Long-term	High
Low	One type of antibody	Transitional	Low

**Table 6:** Indications for the determination of lupus anticoagulant [3].

**Patients <50 years of age**	▪ Unprovoked venous thromboembolism ▪ Ischemic stroke, transient ischemic attack, or other signs of cerebral ischemia ▪ Arterial thrombosis of a different location ▪ Symptoms accompanying antiphospholipid syndrome but not included in the 2023 criteria, e.g., cognitive impairment, or valvular disease with concomitant systemic autoimmune disease ▪ After an episode of provoked venous thromboembolism, if the triggering environmental factor was disproportionately mild
**Venous** **thromboembolism** **in an unusual location**	▪ Mainly cerebral veins and venous sinuses of the meninges ▪ Retinal veins ▪ Splanchnic veins ▪ Portal vein ▪ Hepatic veins (Budd–Chiari syndrome) ▪ Mesenteric veins ▪ Renal veins
**Thrombosis of small vessels (microthrombosis)**	
**Recurrent venous** **thromboembolism**	▪ Which cannot be attributed to cancer, insufficient anticoagulation, or lack of compliance with recommendations
**Obstetrical failure**	▪ Fetal loss after 10 weeks of gestation, recurrent early miscarriage (first trimester) ▪ Premature birth (before 34 weeks of gestation), with associated preeclampsia or eclampsia, ▪ HELLP (hemolysis, elevated liver enzymes, and low platelet count) syndrome, placental insufficiency (fetal growth restriction), or stillbirth
**Systemic lupus erythematosus**	
**Immune** **thrombocytopenia**	▪ When accompanied by inflammation, joint pain, hair loss, sun sensitivity, oral ulcers, skin rash, or venous thromboembolism
**Livedo reticularis**	▪ When accompanied by another systemic autoimmune disease or moderate thrombocytopenia
**Unexplained, coincidentally detected prolongation of activated partial thromboplastin time (APTT)**	

**Table 7:** Recommendations for primary prevention of antiphospholipid syndrome (APS) in adult patients [40].

High-Risk Profile of Antiphospholipid Antibodies	Systemic Lupus Erythematosus Patients Without Clinical Symptoms		Non-Pregnant Women with a History of Obstetric Antiphospholipid Syndrome
	High-Risk Profile	Low-Risk Profile	Moderate or High-Risk Profile
Low doses of acetylsalicylic acid (75–100 mg/day)	Low doses of acetylsalicylic acid (75–100 mg/day) or hydroxychloroquine	Low doses of acetylsalicylic acid (75–100 mg/day) should be considered	Low doses of acetylsalicylic acid (75–100 mg/day) should be considered after risk assessment

**Table 8:** Recommendations for secondary prevention of antiphospholipid syndrome (APS) in adult patients [40,53].

**Patients with confirmed APS and a first episode of venous thromboembolism**	▪ VKA treatment is recommended (INR 2.0–3.0) ▪ Rivaroxaban should not be used in patients with triple-positive aPL (increased risk of thrombosis) ▪ DOAC may be considered in patients with difficulties in maintaining an appropriate INR or contraindications to VKA ▪ In patients with a first, unprovoked episode of VTE, anticoagulation should be continued long-term ▪ In patients with a provoked first episode of VTE, the duration of treatment should be in accordance with general recommendations; longer treatment should be considered in patients with a persistent high-risk profile or other risk factors for recurrence
**Patients with confirmed APS and recurrence of venous thromboembolism despite VKA treatment (INR 2.0–3.0)**	▪ Consider checking the patient’s compliance with the recommendations and frequent INR testing ▪ If the desired INR has been achieved, consider adding low doses of ASA or increasing the INR to 3.0–4.0 or changing the drug to LMWH
**Patients with APS and a first episode of arterial thrombosis**	▪ VKA treatment is recommended rather than ASA alone ▪ VKA treatment is recommended (INR 2.0–3.0 or 3.0–4.0), considering the individual risk of bleeding and recurrent thrombosis ▪ Combined treatment with VKA (INR 2.0–3.0) and low doses of ASA can also be considered ▪ Rivaroxaban should not be used in triple-positive patients with arterial thrombosis

Abbreviations: aPL—antiphospholipid antibodies, INR—international normalized ratio, DOAC—direct oral anticoagulants, ASA—acetylsalicylic acid, LMWH—low-molecular weight heparin, VKA—vitamin K antagonist.

**Table 9:** Recommendations for prevention of antiphospholipid syndrome (APS) in women [39,40].

**Catastrophic APS**	▪ It is recommended to use direct oral coagulation inhibitors (rituximab and eculizumab)
**Obstetric APS**	▪ In women with a high-risk aPL profile but without a history of thrombosis and/or obstetric complications, treatment with low-dose ASA (75–100 mg/day) should be considered during pregnancy
	▪ In women with a history of only obstetric APS (without thrombosis) with ≥3 recurrent miscarriages <10 weeks of pregnancy or fetal loss (≥10 weeks of pregnancy), combined use of low-dose ASA (75–100 mg/day) and LMWH in prophylactic doses is recommended during pregnancy ▪ In women with a history of delivery <34 weeks due to severe preeclampsia or severe placental insufficiency, low-dose ASA (75–100 mg/day) alone or combined with LMWH in prophylactic doses is recommended ▪ Extension of LMWH in the postpartum period is recommended

Abbreviations: aPL—antiphospholipid antibodies, DOAC—direct oral anticoagulants, ASA—acetylsalicylic acid, LMWH—low-molecular weight heparin, VKA—vitamin K antagonist.

## Data Availability

No new data were created or analyzed in this study.

## Abbreviations

The following abbreviations are used in this manuscript:

aβ2-GPI	Anti-β2-glycoprotein I antibody
aCL	Anticardiolipin antibody
ACR	American College of Rheumatology
aPL	Antiphospholipid antibody
APS	Antiphospholipid syndrome
APTT	Activated partial thromboplastin time
ASA	Acetylsalicylic acid
ASTRO-APS	Apixaban for Secondary Prevention of Thromboembolism Among Patients with Antiphospholipid Syndrome
BAL	Bronchoalveolar lavage
β2GPI	β2-glycoprotein I
DOAC	Direct oral coagulation inhibitor
dRVVT	Dilute Russell viper venom test
ELISA	Enzyme-linked immunosorbent assay
eNOS	Endothelial nitric oxide synthase
EULAR	European Alliance of Associations for Rheumatology
HELLP	Hemolysis, elevated liver enzymes, and low platelet count syndrome
HLA	Human leukocyte antigen
ICAM-1	Intercellular adhesion molecule 1
IgA	Immunoglobulin A
IgG	Immunoglobulin G
IgM	Immunoglobulin M
IL-8	Interleukin 8
INR	International normalized ratio
ISTH	International Society on Thrombosis and Hemostasis
LA	Lupus anticoagulant
LMWH	Low-molecular-weight heparin
MAPK	Mitogen-activated protein kinase
NET	Neutrophil extracellular trap
NF-κB	nuclear factor kappa B
PAI-1	Plasminogen activator inhibitor-1
PAPS	Primary antiphospholipid syndrome
SAPS	Secondary antiphospholipid syndrome
SCT	Sillica Clotting Time
SIRS	Systemic inflammatory response
SLE	Systemic lupus erythematosus
SN-APS	Seronegative antiphospholipid syndrome
TF	Tissue factor
TFPI	Tissue factor pathway inhibitor
TNF-α	Tumor necrosis factor alpha
VCAM-1	Vascular cell adhesion molecule
VKA	Vitamin K antagonist
VTE	Venous thromboembolism
vWF	von Willebrand factor
